# Increased caries risk in a Xerostomic patient with Darier disease: a case report of incipient and advanced carious lesions

**DOI:** 10.1093/omcr/omaf042

**Published:** 2025-05-28

**Authors:** Kostis Giannakopoulos, Persefoni Lambrou, Eleftherios G Kaklamanos, Maria Nefeli Christodoulou

**Affiliations:** School of Dentistry, European University Cyprus, 6 Diogenes str, 2404 Engomi, Nicosia, Cyprus; Scientific Collaborator, School of Dentistry, European University Cyprus, 6 Diogenes str, 2404 Engomi, Nicosia, Cyprus; School of Dentistry, European University Cyprus, 6 Diogenes str, 2404 Engomi, Nicosia, Cyprus; School of Dentistry, Faculty of Health Sciences, Aristotle University of Thessaloniki, Central Secretariat, 1st Basement, 54124 Thessaloniki 54124, Greece; Hamdan Bin Mohammed College of Dental Medicine, Mohammed Bin Rashid University of Medicine and Health Sciences, Dubai 505055, United Arab Emirates; West Suffolk Hospital: Bury St Edmunds, GB

**Keywords:** Darier disease, xerostomia, white spots, dental caries, carious lesions

## Abstract

Darier disease is a rare autosomal dominant inherited skin disorder, caused by mutations in the ATP2A2 gene. Oral manifestations can include mucosal lesions, salivary gland involvement, and xerostomia, that can significantly increase the risk of dental caries.

A 58-year-old female with a confirmed diagnosis of Darier disease presented with oral manifestations, including recurrent sublingual blisters, xerostomia, and carious lesions. The intraoral examination revealed white spot carious lesions and cavitated caries, particularly in the cervical areas of several teeth. Dental treatment included fluoride mouthrinse, oral hygiene instructions, and restorative care for the cavitated teeth. The patient was also advised on preventive and symptomatic measures for xerostomia.

Dentists should be aware of these potential issues and provide appropriate preventive, etiologic and symptomatic care. Regular follow-ups are essential to monitor the progression of dental lesions and manage oral health in patients with Dd.

## Introduction

Darier disease (Dd), also known as Darier—White disease or keratosis follicularis, was independently described by Darier and White in 1889 [1, 2]. Dd is a rare autosomal dominant inherited skin disorder, with variable expressivity, caused by a gene mutation on chromosome 12q23–24.1 [3] and identified in 1999 as a mutation in ATP2A2, encoding the sarco/endoplasmic reticulum ATPase type 2 (SERCA2), a calcium pump distributed throughout the endoplasmic reticulum [4]. Although inherited, 47% of Dd patients have no family history, likely due to de novo mutations in germ cells or early embryonic development. These mutations are absent in parental somatic cells, making the affected individual the first in their family to develop the disease [5, 6]. Somatic mosaicism can also explain localized or segmental presentations, where post-zygotic mutations lead to a mix of affected and unaffected cells. Earlier mutations result in widespread disease, while later ones cause segmental Darier disease, with lesions following Blaschko’s lines [7, 8].

Prevalence is reported to range from 1 in 36 000 to 1 in 100 000 [9, 10] or by other authors, similarly, between 1 in 30 000 and 1 in 100 000 [11]. Yeshurun et al. in 2021 estimated a prevalence of approximately 1 in 55 000, though the specific region was not specified [12]. Other studies report prevalence at 1 in 100 000 in Scandinavia, 1 in 36 000 in northeast England, and 1 in 55 000 in Oxfordshire, England, with an estimated incidence of new cases at 4 per million per 10 years [13–15]. Notably, a recent study found a higher prevalence in Sweden, reporting approximately 1 in 12 000 individuals, suggesting that Darier disease may be more common in certain populations than previously thought [16]. Given these figures, Darier disease is classified as a rare disease, where a condition is defined as rare if the prevalence is less than 1 in 2000 people [17]. It is important to note that prevalence and incidence rates for Darier disease are not well-established, and estimates can vary due to factors such as study methodologies and population differences.

Dd predominantly affects seborrhoeic skin areas, such as the forehead, scalp margin, nasolabial folds, ears, chest, and upper back and presents in up to 96% of patients with distinctive nail abnormalities. Lesions often have a malodorous characteristic [18, 19].

Mucosal manifestations were first described by Reenstierna in 1917 [20], and oral involvement is reported in about 50% of Dd cases, with the hard palate being the most common site. Mild forms resemble nicotinic stomatitis, while more severe cases present as papillary palatal hyperplasia [5, 21]. The gingiva is the second most affected site, followed by the buccal mucosa, tongue, and floor of the mouth [22]. Lesions are typically asymptomatic and discovered during routine dental examination, often requiring no treatment [21, 23]. Although oral lesions are not premalignant, there has been a report of oral squamous cell carcinoma in a Dd patient [19].

Salivary gland involvement in Dd includes obstructive sialadenitis and swelling, especially in the parotid and submandibular glands, affecting around 30% of patients [21, 24–29]. Sialographic and histopathologic studies indicate a progressive nature of salivary duct involvement, with chronic inflammation and periductal fibrosis preceding obstruction. The latter includes squamous metaplasia leading to occlusion of the lumen, occurring particularly in the larger ducts [28] or in the vicinity of the ductal orifice [26]. The degree of salivary gland involvement does not correlate with the degree of either skin or mucosal disease [25]. Xerostomia has also been documented [30], including a case with concomitant xerophthalmia and Sjögren’s syndrome [31]. Darier disease can also, although very rarely, give rise to a severe complication, such as Kaposi-Juliusberg syndrome, a disseminated cutaneous Herpes Simplex Virus infection accompanied by generalized signs [28].

Differential diagnosis should include seborrheic dermatitis, Hailey—Hailey disease, Acanthosis nigricans and Epidermodysplasia verruciformis [10].

Treatment for Dd varies, with retinoids (topical and systemic) and fluorouracil being the most effective non-surgical options. Surgical approaches include excision, dermabrasion, and CO2 laser ablation for localized lesions [32]. In all cases, long-term management requires the use of moisturizers, sun protection, and the avoidance of known triggers [33].

## Case report

A 58-year-old female patient presented at the dental clinic of the European University Cyprus expressing concern about oral manifestations of Darier disease (Dd). Her chief complaint was relapsing blisters under the tongue, which she reported would break upon pressure, releasing fluid. Additionally, she was worried about white areas on several of her teeth and cavitations that required dental care.

The patient’s medical history includes a confirmed diagnosis of Dd, which first manifested during puberty with skin lesions on the chest. These lesions persisted throughout her life, varying in severity and affecting areas such as the eyebrows, chest, hands, soles, and other areas, presenting as red papules, with moderate itching, and moderate malodor. The disease also affects her nails. The diagnosis was confirmed histopathologically through a skin biopsy and later by DNA testing. The clinical genetics report confirmed the patient is heterozygous for a likely pathogenic de novo variant in AT2PA2 and also heterozygous for a 0.2 Mb duplication at 12q24.33 of unclear significance, which is present in both her parents who originate from the same village in the country of her residence. The patient has experienced numerous secondary skin infections related to her condition. She is under dermatological and ophthalmological care, the latter due to corneal irritation caused by hyperkeratosis of the eyelashes. Evaluations by neurosurgeons and neurologists at a public General Hospital included a brain MRI, which revealed a longstanding history of a possible cavernous angioma (differential diagnosis: small AVM) and upper lip spasms. The patient also has a history of anxiety disorder and depressive symptoms and is under psychiatric surveillance.

In addition, she reported a history of herpes zoster, which occurred twice. There is no familial history of Darier disease; DNA testing confirmed that her parents and daughters are disease-free.

Regarding past treatments, the patient underwent photodynamic therapy one year ago, which led to severe burn side effects, described by her as nearly fatal, with eventual recovery but no positive therapeutic outcomes. She has a history of receiving acitretin, a second-generation retinoid. Currently, her treatment includes only vitamin A, vitamin D, and omega-3 supplements, alongside the use of sun protectors. The patient does not report any noticeable improvement in xerostomia from the use of omega-3 fatty acid supplementation.

Oral manifestations reported by the patient include recurrent blisters in the sublingual area, which release mucous fluid upon rupture. She also experiences xerostomia, predominantly on the tongue but also throughout the oral cavity, along with a persistent salty taste. Additionally, she reports occasional swelling of the submandibular lymph nodes. Although she underwent an ultrasound a few weeks prior, she did not have the report; she claimed the results were free of pathological findings.

**Figure 1:**
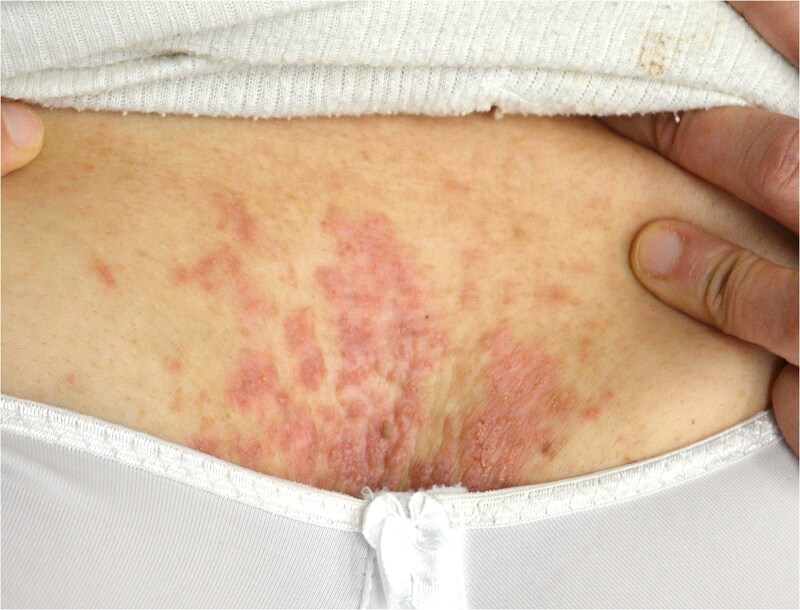
Papules on the chest are characteristic lesions of DD.

**Figure 2:**
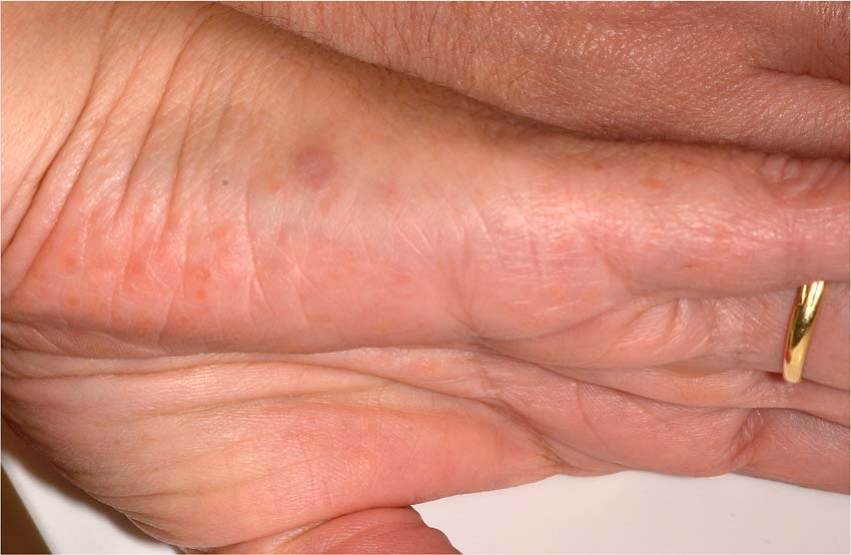
Lesions are visible on the lateral surfaces of the hands.

**Figure 3:**
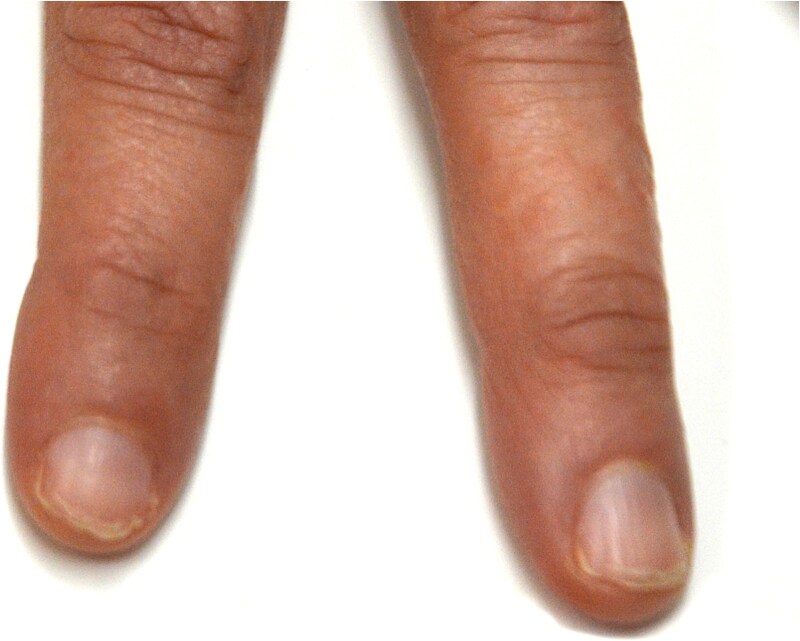
Lesions affecting the nails are evident, showing typical nail involvement in DD.

**Figure 4:**
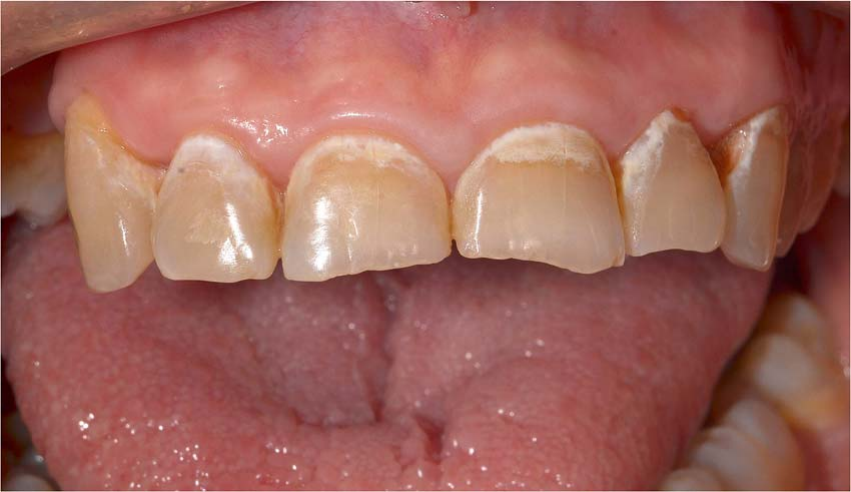
White spot caries lesions are evident on all maxillary anterior teeth.

**Figure 5:**
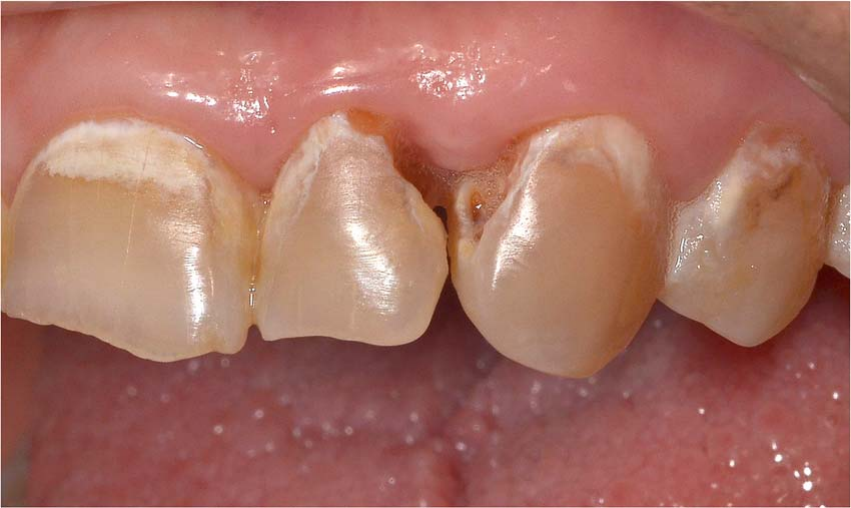
Advanced, cavitated caries lesions are visible on teeth # 22 and 23.

**Figure 6:**
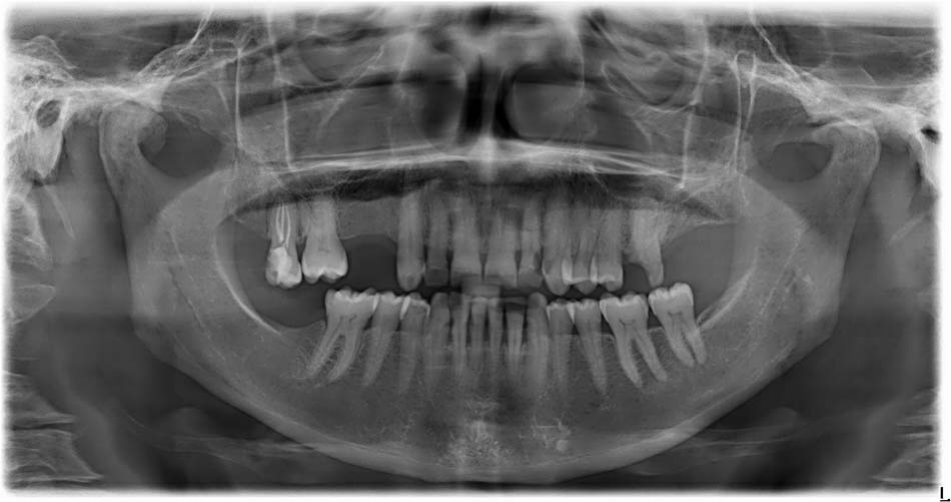
Panoramic radiograph confirms the findings.

The clinical examination revealed characteristic Dd lesions on the chest (Fig. 1), hands (Fig. 2), and nails (Fig. 3). Intraoral examination showed missing teeth #18, 15, 14, 27, 28, 38, 47 and 48, as well as root canal treatment on tooth #27. White spot carious lesions were noted primarily in the cervical areas of teeth #13 to 24, with cavitations evident on teeth #12,22, 23 and 26 (Figs. 4 and 5). A panoramic radiograph was taken, confirming the findings (Fig. 6) and also revealing carious lesions on teeth #33, 41, 42 and 43.

**Figure 7:**
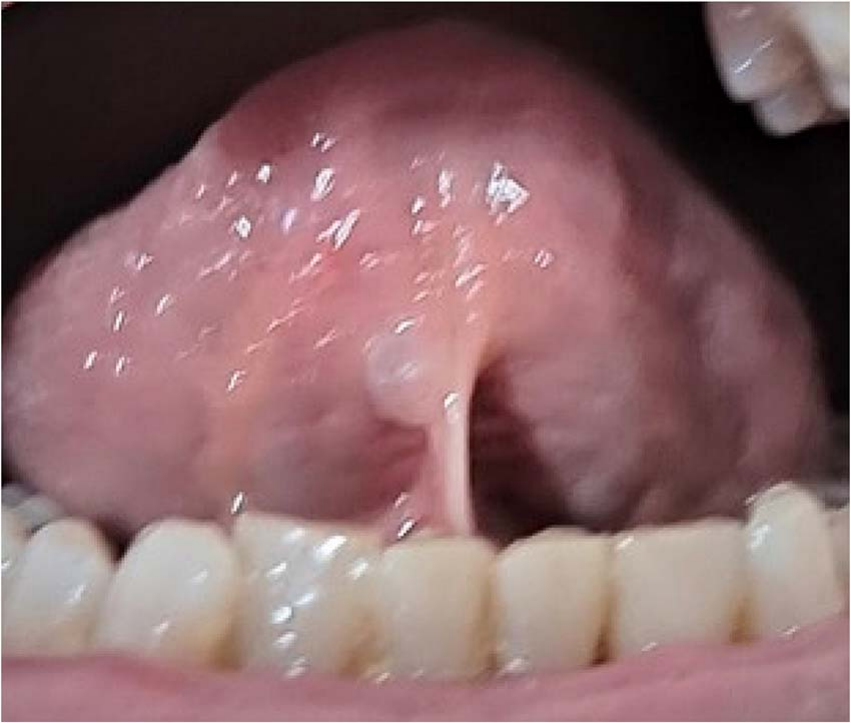
A fluid filled blister in the sublingual area, which ruptures upon pressure.

The patient was advised to use a fluoride mouthrinse, and oral hygiene and dietary instructions were provided. She was scheduled for endodontic and restorative evaluation of tooth #26, professional prophylaxis, and composite resin restorations on the cavitated teeth. Soft tissue examination revealed a blister in the right sublingual area (Fig. 7). The patient declined the replacement of missing teeth. She was provided symptomatic treatment options for xerostomia that included chewing xylitol gum and artificial saliva supplements, when needed. Follow-up will be conducted every six months to monitor the progression of the incipient carious lesions.

## Discussion

Darier disease (Dd) is a non-life-threatening condition that primarily affects the skin and nails, with approximately 50% of cases showing oral manifestations that usually require no treatment [3, 5, 21]. Xerostomia, a condition associated with hyposalivation can result from the malfunction of salivary glands due to systemic or local factors. It frequently occurs in conditions like Sjögren’s syndrome, which involves dry mouth and dry eyes and may co-occur with rheumatoid arthritis or a related connective tissue disease. Other common causes include radiation therapy for head and neck malignancies and local factors such as sialadenitis, sialolithiasis, acute oral infections, neoplasms, and trauma to salivary gland secretory tissue [34, 35]. Commonly prescribed medication is a leading cause, and Acitretin, a systemic retinoid that the patient reported in this article was taking in the past, is known to cause xerostomia due to its effects on mucocutaneous tissues, leading to dryness in the oral cavity, eyes, and skin. Retinoids reduce sebaceous and salivary gland secretion, which can contribute to dry mouth symptoms independently of underlying conditions [36]. Xerostomia has been reported in association with Dd [23, 24], as seen in the present case and while the disease itself has been linked to salivary gland dysfunction, it is important to consider that acitretin might have exacerbated or even primarily contributed to the patient’s dry mouth symptoms. Given the known side effects of acitretin, it is plausible that the patient’s xerostomia persisted or worsened following retinoid therapy.

The patient presented here is also taking omega-3 fatty acids that are known for their anti-inflammatory properties and potential benefits for mucosal health. However, there is currently no direct evidence supporting their efficacy in treating xerostomia associated with Darier disease. Some studies have examined their role in dry mouth conditions like Sjögren’s syndrome and chemotherapy-induced xerostomia, with mixed results [37].

Clinical problems linked to hyposalivation include dental caries, candidiasis, and difficulty in using dentures. Saliva plays a crucial role in maintaining oral health, from moisturizing the mouth and preparing food for swallowing to modulating taste. Importantly, saliva’s buffering capacity helps maintain the pH of the oral environment between 6.8–7.2, key in caries prevention, while its antimicrobial properties due to the lactoferrin and lysozyme are also important. This is why patients with reduced salivary flow are at greater risk of developing caries lesions or experiencing rapid progression of existing ones [38, 39]. In this case, several caries lesions were identified, including incipient and cavitated lesions. The patient’s oral hygiene was moderate, with toothbrushing reported once a day, no flossing, and her diet was moderately cariogenic.

It is crucial for clinicians to identify the underlying cause of xerostomia and provide the patient with appropriate treatment. Whenever possible, treatment should be etiological, but more commonly symptomatic management is required. For drug induced xerostomia, medication adjustments may be necessary, and local factors should be managed accordingly. Key interventions include thorough oral hygiene and dietary instructions, fluoride application through toothpaste and mouthrinse and avoiding dry, acidic, and salty foods as well as tobacco and alcohol [35].

In this case, the patient presented with multiple white spot lesions and cavitated caries that necessitate restorative treatment. The existing xerostomia likely contributes to these caries lesions and possibly to missing teeth. Although xerostomia is reported in Dd, the exact mechanism of hyposalivation remains unclear [30, 31]. Caries risk analysis is vital, and comprehensive preventive measures such as patient education and motivation, oral hygiene guidance, and dietary recommendations should be a priority [40]. Routine fluoride use, xylitol chewing gum and other caries prevention methods should be employed [41]. Frequent recalls are essential to monitor the patient’s condition, allowing for early detection and management of cavities to achieve minimal damage to the teeth.

Additionally, the patient’s history of herpes zoster reported alongside herpes simplex infections in a study by Tegner et al. [28], suggests a possible link with viral complications in Dd.

## Conclusions

Darier disease (Dd) is typically diagnosed by dermatologists, with oral manifestations often identified during routine dental examinations [21, 23]. Oral healthcare providers should be vigilant when treating patients with Dd, as they may present with soft tissue or salivary gland manifestations having an increased risk of dental caries. Although there is no conclusive evidence that gingival involvement in Dd predisposes to periodontitis, or that oral mucosal lesions carry malignant potential, appropriate preventive advice and regular follow-ups are essential for these patients [24]. Additionally, clinicians should be aware of the potential for concomitant Sjögren’s syndrome, as both conditions may cause xerostomia and xerophthalmia [31].

## Consent

Full informed consent was obtained from the patient for the dental examination, radiography, photography and case presentation/publication.

## Guarantor

Dr. Kostis Giannakopoulos is nominated as Guarantor of the paper.
